# Deep-learning-based image reconstruction with limited data: generating synthetic raw data using deep learning

**DOI:** 10.1007/s10334-024-01193-4

**Published:** 2024-08-29

**Authors:** Frank Zijlstra, Peter Thomas While

**Affiliations:** 1 Department of Radiology and Nuclear Medicine, St Olav’s University Hospital, Postboks 3250 Torgarden, 7006 Trondheim, Norway; 2 Department of Circulation and Medical Imaging, NTNU–Norwegian University of Science and Technology, Trondheim, Norway

**Keywords:** Accelerated MRI, Deep learning, Image reconstruction, Synthetic data

## Abstract

**Object:**

Deep learning has shown great promise for fast reconstruction of accelerated MRI acquisitions by learning from large amounts of raw data. However, raw data is not always available in sufficient quantities. This study investigates synthetic data generation to complement small datasets and improve reconstruction quality.

**Materials and methods:**

An adversarial auto-encoder was trained to generate phase and coil sensitivity maps from magnitude images, which were combined into synthetic raw data.

On a fourfold accelerated MR reconstruction task, deep-learning-based reconstruction networks were trained with varying amounts of training data (20 to 160 scans). Test set performance was compared between baseline experiments and experiments that incorporated synthetic training data.

**Results:**

Training with synthetic raw data showed decreasing reconstruction errors with increasing amounts of training data, but importantly this was magnitude-only data, rather than real raw data. For small training sets, training with synthetic data decreased the mean absolute error (MAE) by up to 7.5%, whereas for larger training sets the MAE increased by up to 2.6%.

**Discussion:**

Synthetic raw data generation improved reconstruction quality in scenarios with limited training data. A major advantage of synthetic data generation is that it allows for the reuse of magnitude-only datasets, which are more readily available than raw datasets.

## Introduction

The development of accelerated MR imaging techniques has long been an active field of research, driven by the inherently slow data acquisition in comparison to other modalities. Faster MR imaging can lower the total examination time and thus reduce the cost of MRI examinations and lower patient burden. This is especially important as healthcare costs and the demand for MRI examinations are increasing.

Typically, accelerating MRI involves undersampling k-space, i.e. violating the Nyquist sampling criterion. Numerous techniques have been developed for reconstructing images from undersampled k-space. These range from conventional approaches such as parallel imaging [1, 2], which exploits redundancy in data acquired with multi-channel coil arrays, to iterative approaches such as compressed sensing [3], which exploits spatial redundancy in reconstructed images. More recently, deep learning (DL) has been shown to have great promise in reconstructing undersampled k-space data [4–12].

Whereas previous approaches require great care and engineering to use redundancy in the acquired data to push towards greater acceleration factors, deep learning is primarily data-driven and can discover such redundancy automatically, based on sufficient training data. Furthermore, the types of redundancy learned will be specific for the training data, e.g. it can be specific for scan type, anatomy, acceleration factor, and coil geometry, among others. By utilizing more sources of redundancy, both acceleration factors and image quality can be substantially improved using deep learning [9, 11, 12].

This improved performance comes at the cost of requiring large amounts of training data. For most end-to-end deep learning reconstruction methods, the training data needs to be fully sampled raw k-space data (i.e. complex-valued data, separated per receive channel). However, this kind of raw data is not routinely stored during clinical examinations or research studies. Acquiring raw data specifically for training DL image reconstruction methods is time-intensive, costly, and subject to ethical approval. Conversely, magnitude-only reconstructed images are more readily available, both in clinical and research settings.

The largest and most frequently used repository of raw data is the FastMRI dataset [13], which provides fully-sampled raw data for over 6000 brain scans and 2000 knee scans. This dataset has proven itself invaluable for the development of DL-based MR image reconstruction [7]. However, with a primary focus on one set of data, there is a risk of poor generalization. First, while FastMRI contains a variety of scan types, these still represent a relatively narrow selection of the common MR imaging protocols and anatomies of interest. Multiple studies have shown that models trained on one anatomy perform poorly on different anatomies [14–16]. Second, when training on a large dataset (e.g. 100s or 1000s of scans) the risk of overfitting to the training data is small, whereas this risk is substantial when training on smaller datasets (e.g. 10s of scans) [17]. As such, methods developed solely on the FastMRI dataset may have poor performance in applications where training data is more limited.

Commonly used methods for mitigating the risks of limited training data are data augmentation [17, 18] and transfer learning [19–21]. Data augmentation uses basic image manipulations (e.g. rotations) on existing training data to increase the effective amount of training data [18], which has been shown to improve model performance in deep learning, both generally [18], as well as specifically for MR image reconstruction [17]. Transfer learning attempts to re-use larger datasets containing different scan types and/or data for different anatomies. A common approach is to pre-train a reconstruction model using the larger dataset and fine-tune it on the limited dataset [20, 21]. However, both data augmentation and transfer learning still require raw data for MR image reconstruction training tasks. As an alternative, Korkmaz et al. proposed learning a deep MR image prior using coil-combined complex-valued reconstructed images [22]. However, these complex-valued reconstructions are not commonly stored either.

Despite the availability of magnitude-only images, only a few studies have studied the re-use of magnitude-only images for training DL-based image reconstruction models that would otherwise require raw, complex-valued, multi-coil k-space data [4, 6, 23–25]. These will be discussed in the following sections. The main goal of this study is to explore the impact of limited availability of raw k-space data for DL-based MR image reconstruction and to investigate avenues to mitigate the lack of training data. We hypothesize that by using deep learning to synthesize synthetic raw training data from magnitude-only images, the size of training sets can be expanded by leveraging existing magnitude-only datasets. Although magnitude data is sometimes used directly for training DL-based image reconstruction, naïve use can lead to bias and poor generalization [25, 26]. A more comprehensive approach is necessary to ensure that the generated synthetic raw data is realistic enough such that a reconstruction model trained on synthetic raw data generalizes well to reconstructing real, undersampled raw k-space data.

In other fields, deep-learning-based synthetic data has shown promising results in mitigating the scarcity of training data where the acquisition of training data is time-consuming, expensive, and/or difficult [27–32]. Such generated data can greatly increase both the amount of training data and the variation in existing data, and is expected to become more common in training deep neural networks as generative deep learning models become more advanced [32].

DL-based generation of synthetic magnitude-only MRI data has been investigated in a number of image processing tasks, such as segmentation [33, 34] and T2 mapping [35], and for general purpose use [36, 37]. Most of these approaches are based on generative adversarial networks (GANs) [38], which aim to produce images indistinguishable from real data, but which may be prone to various kinds of artifacts [39, 40]. Here, it is important to note that these examples generated magnitude-only, reconstructed images, and not synthetic raw data.

Several studies have investigated generating raw MRI data (i.e. complex-valued data separated by receive coil) without the use of deep learning, for example through generating sinusoidal phase maps and reusing coil sensitivity maps from other scans [4, 6, 24]. In contrast, few studies have investigated DL-based generation of raw MRI data for image reconstruction [23, 25]. During the preparation of this manuscript, one study was published with the same goal that we pursued in this work, i.e. generating raw MRI data from magnitude-only data. Deveshwar et al. [25] investigated generating phase information from magnitude-only images using the pix2pix conditional GAN [41], and showed that DL-based generation outperformed other methods of synthetically generating phase information in various reconstruction tasks. However, the generated phase maps did show artifacts that the authors attributed to the PatchGAN discriminator network [25, 41]. Furthermore, the study did not generate coil sensitivity maps (CSM) and noted that attempting multi-coil generation resulted in severe image artifacts [25].

In this study, we utilized adversarial autoencoders (AAEs) [42] for generating synthetic phase and coil sensitivity information from magnitude-only images. In contrast to GANs, AAEs learn to represent their training data in a latent space, which can be sampled to generate new data points. Importantly, a conditional AAE can generate multiple outputs from the same input, whereas the pix2pix conditional GAN will always produce the same output for a given input [41]. This is important, because the magnitude-to-phase/CSM transformation problem is ill-posed, i.e. there are many plausible outputs for one input. Using a model that can represent this variability enables sampling multiple, plausible phase/CSM maps from a single magnitude image, and thus allows a larger amount of synthetic raw data to be generated. Furthermore, the adversarial loss of an AAE is only applied to the latent space and only backpropagates into the encoder network, and thus reduces the likelihood of image artifacts that may arise from the image-based adversarial losses used in GANs [25, 39, 40].

## Materials and methods

This study evaluated the effectiveness of synthetic raw data generation on a fourfold accelerated deep learning MR reconstruction problem with increasing amounts of training data. By observing differences in reconstruction quality, measured by multiple metrics, we assessed the efficacy of synthetic raw data in mitigating the loss of reconstruction quality associated with training DL-based MR reconstruction networks on small datasets. The following sections describe the training data, reconstruction, and synthetic data generation methodology in more detail.

### Data

For this study, we selected a single T1-weighted GRE scan type from the FastMRI dataset [13], where all scans had identical scan parameters: flip angle 70, TE 2.64 ms, TR 250 ms, TI 300 ms, matrix size $16\times 320\times 320$. These scans were acquired on two scanners (Skyra and Prisma fit, Siemens, Erlangen, Germany) at a field strength of 3 T. The images were acquired with either 16-channel head arrays or 20-channel head-and-neck arrays, where for the latter we used only the data from the 16 channels around the head.

This yielded 184 scans, which were split into a training set of 160 scans, a validation set of 4 scans, and a test set of 20 scans. Scans were balanced to maintain a consistent distribution of scanner hardware and receiver arrays in each set.

The raw data of each scan was reconstructed using a phase-sensitive coil combination technique [43], which yielded complex-valued reconstructions and complex-valued coil sensitivity maps (CSM). To keep the coil sensitivity maps consistent across scans, we divided all CSMs by the average phase of the CSM of the first channel. The raw data was scaled with a constant factor of 10^6^ to obtain reconstructions normalized around unit intensity. No normalization per subject was performed because we found the original scaling in the dataset to be appropriate. For each scan, the noise covariance in the raw data was calculated from a $20\times 20$ region that was manually selected in the background signal.

### Reconstruction

For all deep learning image reconstruction experiments, we used the end-to-end Variational Network [44] without modification. This network was chosen based on its state-of-the-art performance, as well as its use as a baseline measure in the FastMRI challenge [9]. Reconstruction was applied to each 2D slice of a dataset individually. We used the retrospective ~ fourfold undersampling scheme that was used in the FastMRI challenge [13]. This samples every 4th line in the phase-encoding dimension of k-space, as well as sampling 26 central lines for coil calibration.

To keep a constant training time among experiments with different training set sizes, we defined one epoch as training on 200 training batches with a batch size of 1, and we trained for a total of 1000 epochs. We used the validation set to assess that the convergence of the model training was adequate.

In the experiments that included data augmentation, we applied the following augmentations with 50% probability of each individual augmentation, and uniform probability of any associated parameters: integer pixel translation (− 8 to + 8 pixels), 90-degree rotations (0, 90, 180, 270 degrees), image mirroring (x-axis mirrored, y-axis mirrored, or both mirrored), rotation (− 10 to 10 degrees), zoom (90–110%), and sub-pixel translation (− 16 to + 16 pixels). We did not explore optimization of these augmentation parameters.

### Synthetic raw data generation

In this study, we developed a DL methodology to generate synthetic raw data from magnitude-only reconstructed images. Instead of using DL to transform magnitude images straight to raw data, we considered MR imaging physics and transformed the magnitude images into phase and coil-sensitivity maps first. These maps represent two mostly independent sources of variance in raw data that are missing in magnitude-only images. Together with the magnitude images, these maps were then used to synthesize raw data. It is important to note that this transformation is not a one-to-one relationship, since for any given magnitude image there can be many different plausible phase and coil-sensitivity maps that could be generated.

In this section, we summarize the methodology to transform magnitude images into phase and coil-sensitivity maps. More complete implementation details are given in Appendix A. The following explanation uses the generation of phase maps as an example but applies identically to coil-sensitivity maps.

**Fig. 1:**
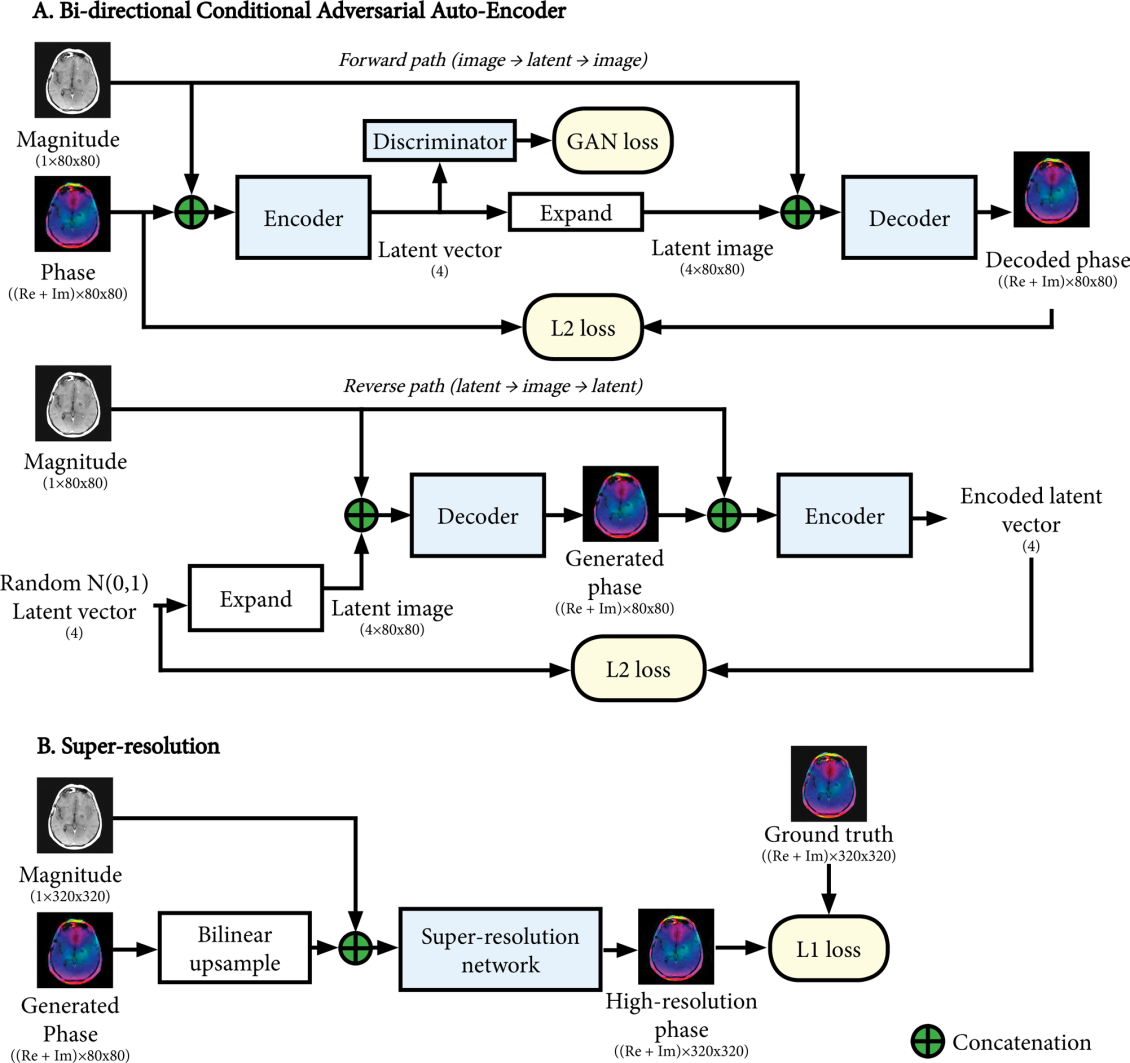
Overview of the bi-directional conditional adversarial auto-encoder (**A**) and super-resolution (**B**) architectures used for generating complex-valued phase maps. The same architectures apply to generating coil sensitivity maps (not shown), with the difference that the maps are 32 channels wide (16x(Re + Im)). The forward path of the auto-encoder encodes a phase map into a low-dimensional representation (4 element latent vector) and is trained to minimize the difference between the decoded phase map and input phase map. A generative adversarial network (GAN) was applied on the latent vector to minimize differences between encoded latent vectors and randomly generated Gaussian latent vectors. The reverse path decodes a random latent vector into a phase map and is trained to minimize the difference between the encoded latent vector and input latent vector, to ensure the latent vector can be recovered from the phase map, and thus promote a one-to-one relationship between the latent vector and generated phase map. Details of the networks used are shown in Fig. 8

The basis of the magnitude to phase transformation is a bi-directional conditional adversarial auto-encoder (AAE) network, an extension of the AAE architecture in Makhzani et al. [42], and is shown in Fig. 1A. This is a combination of an encoder network and a decoder network designed to find a low-dimensional representation of the phase information in a so-called latent space. The encoder takes a magnitude image and ground truth phase image and produces a latent vector. The decoder takes a magnitude image and latent vector and produces a phase image. The networks are trained to minimize the difference between the output and the ground truth phase image.

The adversarial nature of this approach ensures that the latent vector behaves such as Gaussian noise, and therefore new phase images can be generated by randomly sampling latent vectors from a normal distribution and applying only the decoder network. As such, for a given magnitude image, this approach can generate many different phase images, where the variability the model can provide is limited by the dimensionality of the latent space.

To improve the training stability of the AAE networks, this approach was applied at a low resolution (fourfold downsampled images, $80\times 80$ voxels). Subsequently, a super-resolution network was trained to upsample the low-resolution synthesized maps back into the original resolution, using the high-resolution magnitude image as an additional input. This process is shown in Fig. 1B.

With auto-encoders trained to generate phase ($P$) and coil-sensitivity maps ($CSM$), raw data can be synthesized by simply multiplying the magnitude image ($M$) with the complex-valued phase map (normalized to unit intensity) and the coil-sensitivity map, and adding Gaussian noise according to known noise-covariance ($\varphi$):

$$raw=M\frac{P}{\left|P\right|}CSM+N(0,\varphi )$$

**Fig. 2:**
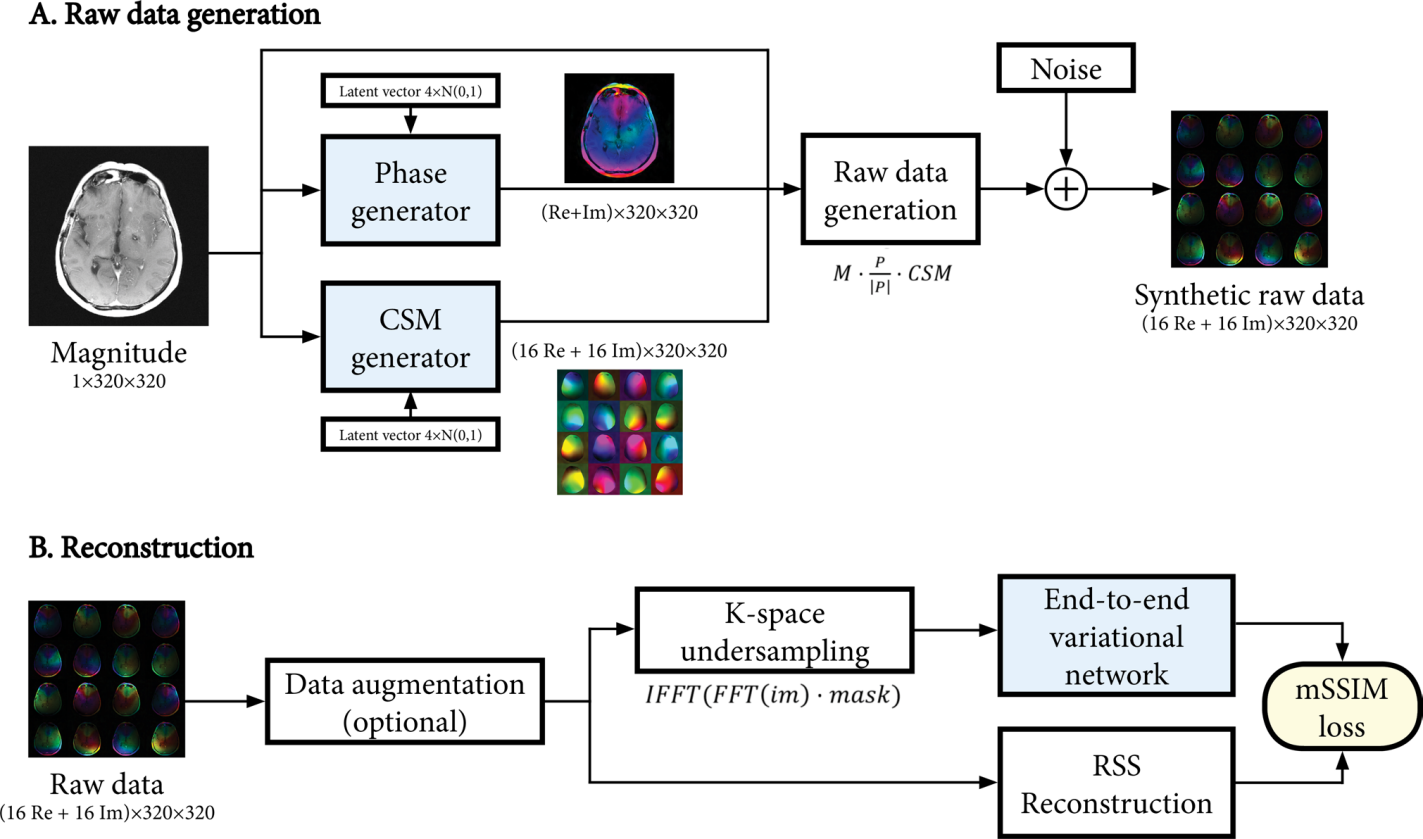
Overview of the synthetic raw data generation (**A**) and image reconstruction (**B**) architectures. The auto-encoder architectures (Fig. 1) are used to generate phase and coil sensitivity maps (CSM) from a magnitude image, which are multiplied with the magnitude image to generate synthetic raw data. Noise is added given a known noise covariance. The reconstruction pipeline is trained using fully sampled raw data (real and/or synthetic), with optional data augmentation (Sect. "Synthetic raw data generation"). Retrospective undersampling is applied in k-space with a given undersampling mask (fourfold regular undersampling) to simulate the undersampled raw data input to the end-to-end variational network. The variational network is trained to maximize the mean structural similarity index measure (mSSIM) between the DL reconstruction from undersampled data and the ground truth root-sum-of-squares (RSS) reconstruction from the fully sampled data

Here, $P$ and $CSM$ are the outputs of the auto-encoder networks, given a latent vector randomly sampled from a normal distribution. The noise covariance was randomly selected from one slice from one of the raw training datasets. The raw data generation process is shown in Fig. 2A. Since applying these models is computationally cheap, new synthetic raw data was generated on-the-fly while training our reconstruction networks (Fig. 2B).

### Experimental setup

For this study, we performed 4 experiments in which reconstruction networks were trained with different training data:

1. Baseline: Training with real raw data without data augmentation
2. Baseline with data augmentation: Training with real raw data with data augmentation
3. Synthetic data: Training with a mix of synthetic raw data and real raw data.
4. Synthetic data with data augmentation: Like the synthetic data experiment, but combined with data augmentation of both the synthetic and real raw data

Each reconstruction experiment was repeated for 4 different amounts of training scans: 20, 50, 100, and 160 scans. In the experiments without synthetic data, these training scans were all sourced from real raw data. In the synthetic data experiments, these different amounts refer to the number of unique magnitude-only data used in training.

In addition, in the synthetic data experiments, real raw data for the first 20 scans of the training set was available to train the generative AAE networks for generating the synthetic raw data. The raw data from these scans were then also included during training of the reconstruction network, in addition to the magnitude-only data. For example, the experiment using 50 scans would use both the real raw data and magnitude-only data of 20 scans, plus magnitude-only data from 30 additional scans. As such, during training of the reconstruction network, two separate pools of raw data were used: 1. an effectively infinite pool of synthetic raw data generated on the fly from the magnitude-only images (i.e. 20, 50, 100, or 160 scans), and 2. a limited set of slices sampled from the same 20 real raw datasets that had been used to train the AAE networks. Based on initial empirical findings, 75% of the training samples were drawn from the synthetic raw data pool and 25% of the training samples were drawn from the real raw data pool.

Each experiment was repeated 5 times to account for variability and randomness in the training process. Each training run in each experiment was evaluated on the test set using mean absolute error (MAE), root mean squared error (RMSE), and mean structural similarity index measure (mSSIM) [45], calculated between the reconstructed image and the ground truth. The ground truth was a root-sum-of-squares reconstruction of the fully sampled data. Out of these 5 repetitions, we selected the network with the best mSSIM performance on the validation set for comparison among the different reconstruction experiments and for visualization of reconstructed images. Paired t-tests (paired by test subject) were performed between the synthetic data experiments and the baseline experiments, using the selected networks.

### Ablation study

Because our synthetic data generation strategy involves generating both phase maps and coil sensitivity maps using deep learning, we performed an ablation study to test the relative importance of including these components. We tested the performance of replacing one or both of these maps with the real, ground-truth maps in the reconstruction experiments. In addition, we tested against the use of sinusoidal phase patterns as described by Zhu et al. [4].

The following combinations were included in this experiment:

1. Real phase maps and real coil sensitivity maps (CSMs)
2. Real phase maps and synthetic CSMs
3. Sinusoidal phase maps and synthetic CSMs
4. Synthetic phase maps and real CSMs
5. Synthetic phase maps and synthetic CSMs (proposed approach)

Here, the first experiment attempts to recreate the baseline real raw data using the synthetic data pipeline, and the final experiment is the synthetic data generation strategy as proposed in this manuscript.

The training strategy and evaluation were performed as described in Sect. "Experimental setup".

## Results

### Synthetic data generation

**Fig. 3:**
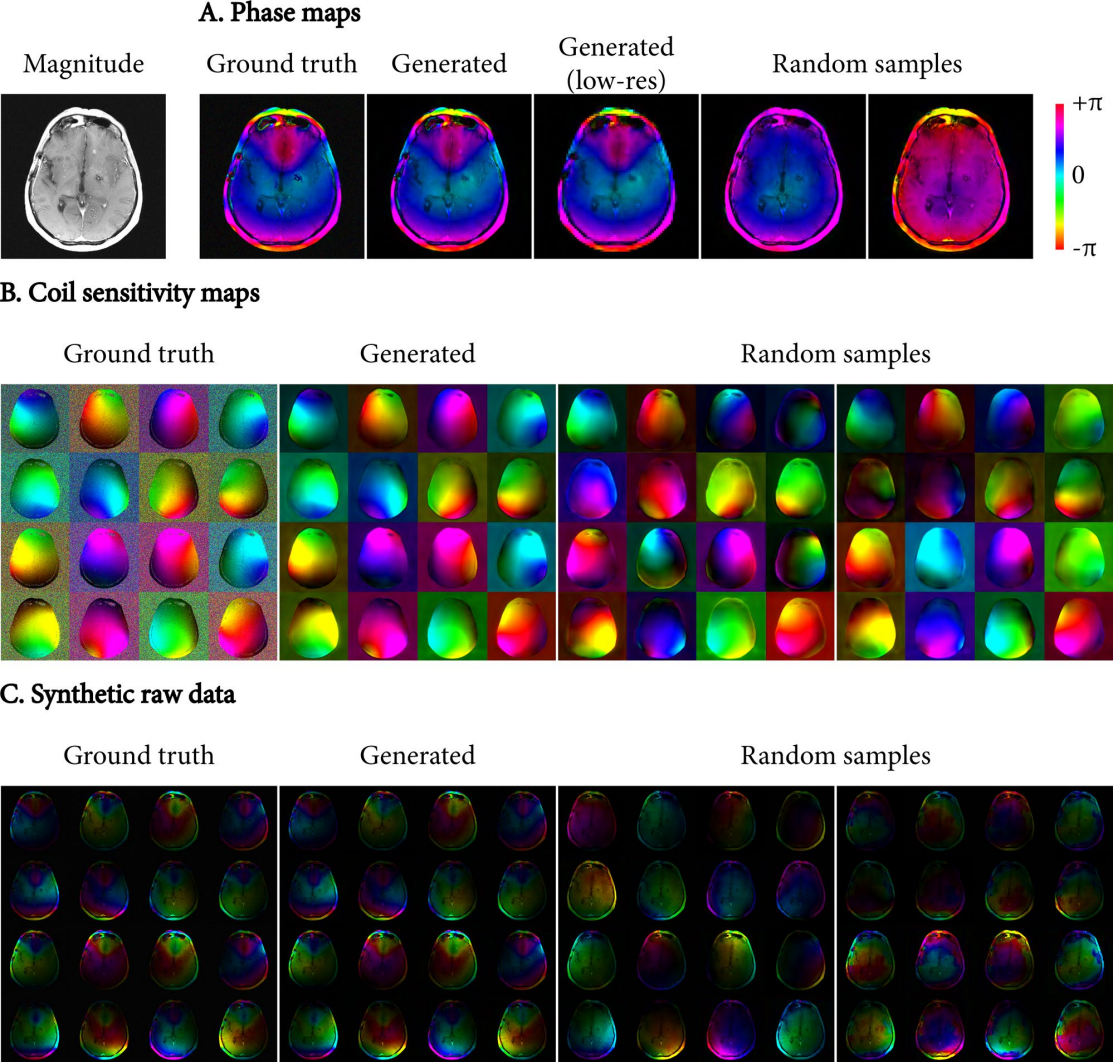
Synthetic phase (**A**) and coil sensitivity maps (**B**) generated from a single magnitude image (top left), and combined into synthetic raw data (**C**). The maps labelled “Generated” were obtained by applying the full auto-encoder to the ground-truth map, demonstrating how well the networks can represent the phase and coil sensitivity information in the low-dimensional latent space. A generated low-resolution phase map is also shown for comparison (i.e. the output of the auto-encoder, prior to the super-resolution network). In addition, two sets of maps generated from a random latent vector are shown, demonstrating the capability of the networks to synthesize new maps. The complex-valued maps are visualized as colour images, where colour represents the phase, and the intensity represents the magnitude of the maps. Additional ground truth images and generated maps are provided in Fig. 4 to illustrate the variability in each

Figure 3 shows examples of phase and coil sensitivity maps generated by the conditional auto-encoder networks. One set of generated phase, CSM, and synthetic raw data uses the full auto-encoder to regenerate the ground truth data (i.e. encoding the ground truth into a latent vector and decoding the latent vector into generated maps). This demonstrates that the networks are able to accurately represent the phase and coil sensitivity information in the limited latent vector. In addition, we show two sets of maps generated from a randomly sampled latent vector. These examples demonstrate the variability sampled by the auto-encoders.

**Fig. 4:**
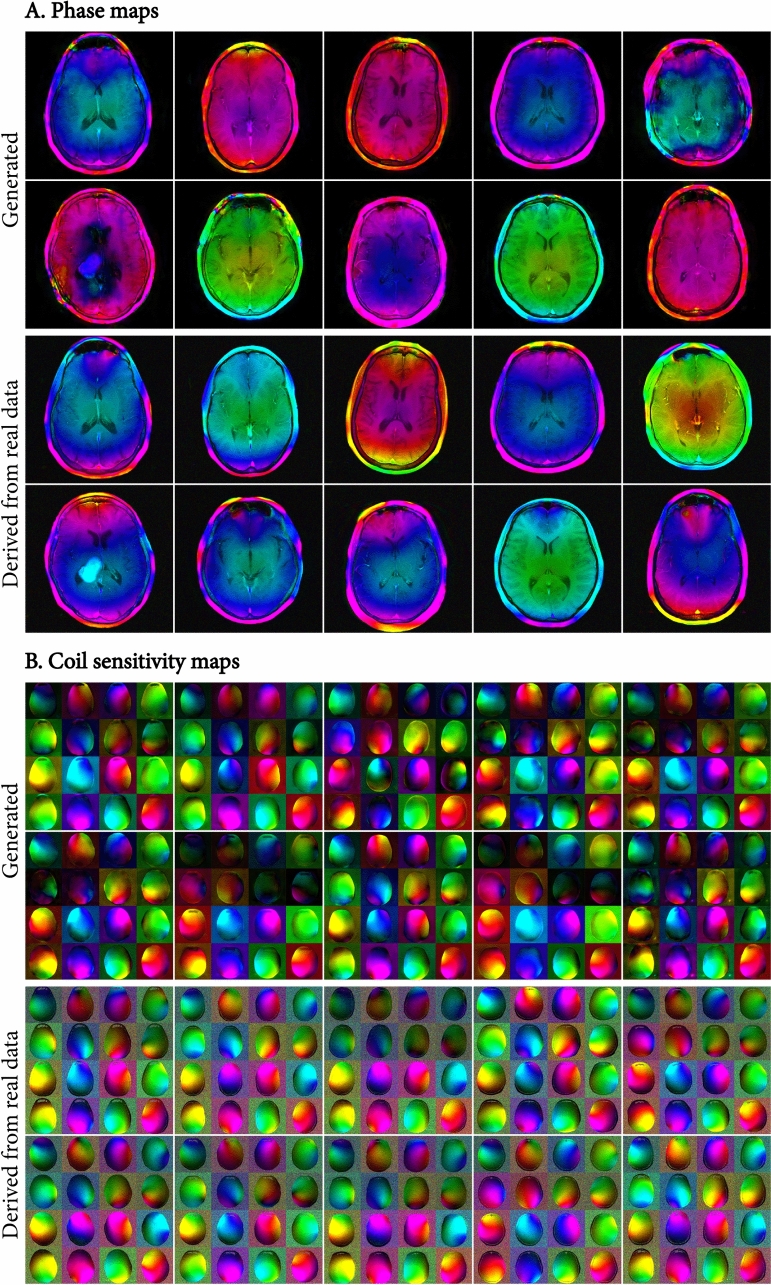
Ten generated phase (**A**, 1st and 2nd row) and coil sensitivity maps (**B**, 1st and 2nd row), and maps derived from real raw data (A/B, 3rd and 4th row) for ten different slices of the test set. Different random latent vectors were used for each of the generated maps. Note that the generated and real maps are not pairs, despite being associated with the same magnitude images. The generated maps were generated randomly with plausible variability, and thus are not expected to produce the same maps

Figure 4 shows additional generated maps and similar maps derived from real raw data. It is important to note that the generated and real maps are not pairs, despite being associated with the same magnitude images. That is, the goal of the randomly generated phase and coil sensitivity maps was not to reproduce the ground truth, but merely to produce a plausible sample with plausible variability. We found the variability in the randomly generated maps to be similar to the variability in the real data. In the generated phase maps, we observed that a small number of maps (2 out of 12 shown in Fig. 3 and 4) showed less realistic, discontinuous phase patterns.

### Image reconstruction

**Fig. 5:**
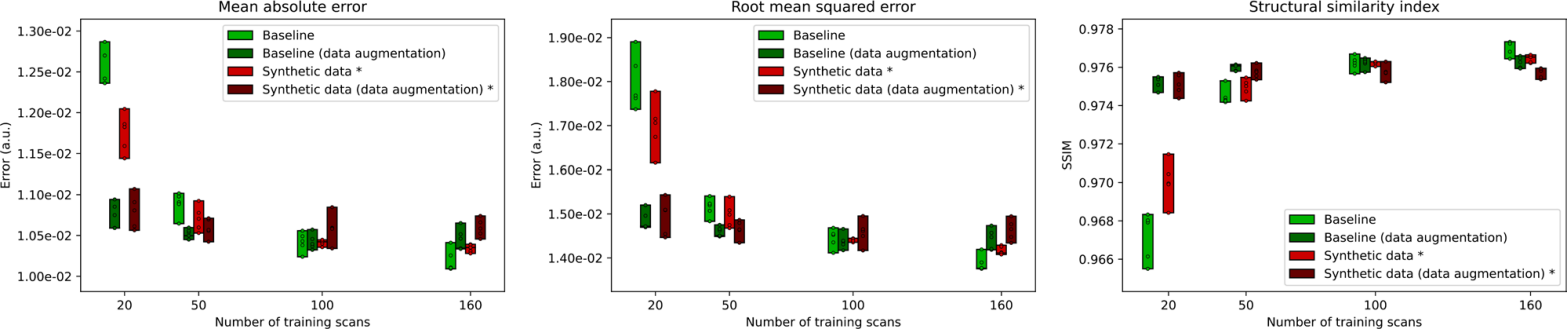
Global reconstruction errors on the test set for the baseline and synthetic data experiments (with and without data augmentation) with increasing amounts of training data, measured by mean absolute error (MAE, left), root mean squared error (RMSE, middle) and mean structural similarity index (mSSIM, right). Each bar displays the range between best and worst results for 5 training runs, with individual runs shown with O marks. Important to note is that the variable amounts of training data for the synthetic data experiments were magnitude-only images (marked with *) in addition to real raw data from 20 images (e.g. the experiment with 160 scans used 20 real raw images and 140 magnitude-only images, see Sect. "Experimental setup"). In contrast, the baseline experiments used variable amounts of real raw data

Table 1 and Fig. 5 show the results of the image reconstruction experiments using real data (baseline) and synthetic data as training data. Table 1 contains the mean reconstruction metrics and standard deviation over all test subjects for the runs with the best validation performance, and Fig. 5 shows the mean reconstruction metrics for all 5 runs. In the baseline, we observe a clear improvement in reconstruction quality with increasing amounts of training data, at least up to 160 scans (i.e. the maximum available in this study). For small training set size, data augmentation showed a substantial improvement (14.4% lower MAE at 20 scans and 1.4% lower MAE at 50 scans). However, with more data, this difference diminishes and at 160 scans data augmentation became a detriment to reconstruction quality (2.5% higher MAE).

**Table 1:** Reconstruction metrics for the image reconstruction experiments (mean ± standard deviation over all 20 test subjects) trained with increasing amounts (20, 50, 100, and 160 scans) of real raw data (baseline) or synthetic data generated from magnitude-only images (marked with * to indicate this difference in data type), both with and without data augmentation

Metric/Experiment	Training scans			
	20	50	100	160
**MAE**				
Baseline	0.0124 ± 1.34e-3	0.0106 ± 1.11e-3	0.0102 ± 1.20e-3	0.0101 ± 0.99e-3
Baseline (data augmentation)	0.0106 ± 0.98e-3	0.0105 ± 0.98e-3	0.0103 ± 0.96e-3	0.0103 ± 0.96e-3
Synthetic data *	0.0114 ± 1.15e-3^†^	0.0105 ± 1.00e-3^†^	0.0104 ± 0.99e-3^‡^	0.0104 ± 0.97e-3^‡^
Synthetic data (data aug.) *	0.0106 ± 0.96e-3	0.0104 ± 0.97e-3^†^	0.0103 ± 0.92e-3	0.0105 ± 0.97e-3^‡^
**RMSE**				
Baseline	0.0174 ± 2.63e-3	0.0148 ± 2.07e-3	0.0141 ± 1.94e-3	0.0138 ± 1.74e-3
Baseline (data augmentation)	0.0147 ± 1.80e-3	0.0145 ± 1.74e-3	0.0142 ± 1.58e-3	0.0142 ± 1.56e-3
Synthetic data *	0.0162 ± 1.86e-3^†^	0.0147 ± 1.85e-3	0.0144 ± 1.82e-3^‡^	0.0141 ± 1.49e-3^‡^
Synthetic data (data aug.) *	0.0145 ± 1.48e-3^†^	0.0143 ± 1.52e-3^†^	0.0142 ± 1.43e-3	0.0143 ± 1.45e-3^‡^
**SSIM**				
Baseline	0.968 ± 7.48e-3	0.975 ± 6.00e-3	0.977 ± 5.89e-3	0.977 ± 5.76e-3
Baseline (data augmentation)	0.976 ± 6.71e-3	0.976 ± 6.37e-3	0.976 ± 6.14e-3	0.977 ± 5.97e-3
Synthetic data *	0.971 ± 6.98e-3^†^	0.975 ± 6.28e-3	0.976 ± 6.08e-3^‡^	0.977 ± 5.89e-3^‡^
Synthetic data (data aug.) *	0.976 ± 6.41e-3	0.976 ± 6.19e-3	0.976 ± 6.34e-3^‡^	0.976 ± 6.57e-3^‡^

In comparison to the corresponding baseline (with or without data augmentation), values marked with † were significantly better (*P* < 0.05) and values marked with ‡ were significantly worse (*P* < 0.05), in a paired t-test (paired per test subject)

The synthetic data experiments showed equal or significantly better (*P* < 0.05) performance than the corresponding baseline experiment for small training set size (7.5% / 0.3% lower MAE at 20 scans and 1.1% / 0.7% lower MAE at 50 scans (without / with data augmentation)) and equal or significantly worse (*P* < 0.05) performance than the corresponding baseline for large training set size (1.8% / 0.2% higher MAE at 100 scans and 2.6% / 1.1% higher MAE at 160 scans (without / with data augmentation)). Here, it is important to note the difference in training datasets: the baseline experiments used only real raw data, whereas the synthetic data experiments used increasing amounts of magnitude-only data in addition to a small set of real raw data. As such, the lower performance for larger training set size was expected, and this is an indication that synthetic raw data was not entirely equivalent to real raw data.

We observed that training with increasing amounts of magnitude images improved reconstruction quality of the synthetic data experiment by at least up to 160 images, though at a slower rate than the baseline trained entirely on real raw data.

The synthetic data experiments were comparable to the baseline experiment with data augmentation for 50 training scans and above. Using data augmentation in addition to synthetic data generation yielded a small improvement at small amounts of data (7.7% lower MAE at 20 scans and 1.0% lower MAE at 50 scans), but no difference with more training data (less than 0.1% MAE difference at 100 and 160 scans).

The results among all 5 runs are generally consistent with the observations in Table 1, although we note that the variability among runs can be substantial, resulting only from the randomness of the training process. In the baseline experiments, for most training set sizes the variability among each set of 5 runs was slightly reduced by using data augmentation. In the synthetic data experiments, using a large training set size (100 and 160 scans) resulted in a substantially lower variability among the 5 runs, but only when not using data augmentation.

**Fig. 6:**
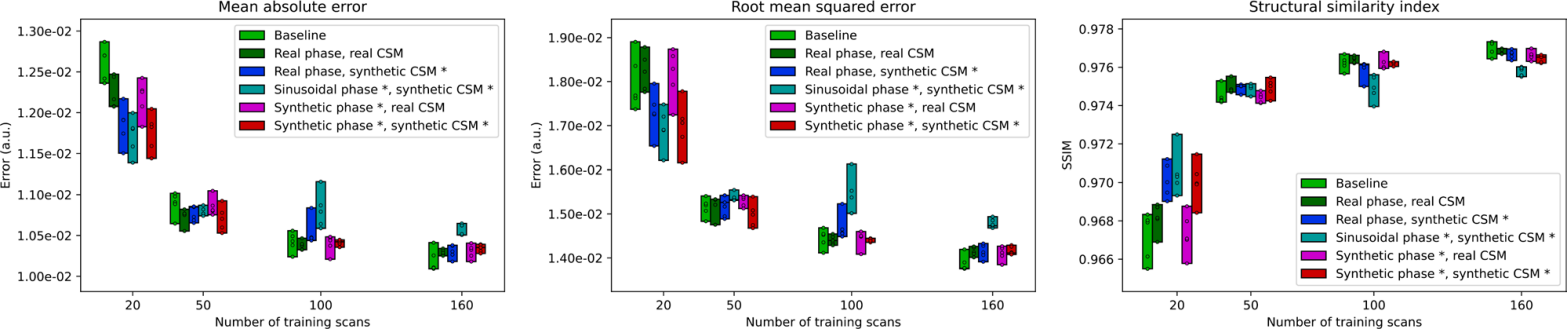
Global reconstruction errors on the test set for the baseline and synthetic data experiments using combinations of different real and/or synthetic phase and coil sensitivity maps (CSMs) to generate synthetic raw data. Results are shown for increasing amounts of training data, and measured by mean absolute error (MAE, left), root mean squared error (RMSE, middle) and mean structural similarity index (mSSIM, right). Each bar displays the range between best and worst results for 5 training runs, with individual runs shown with O marks. Important to note is that the variable amounts of training data for the synthetic maps were magnitude-only images (marked with *, see Sect. "Experimental setup"), whereas the real maps were derived from real raw data

Table 2 and Fig. 6 show the results from the ablation experiment, using different combinations of real, sinusoidal, and synthetic phase maps, and real and synthetic coil sensitivity maps (CSM) to generate synthetic raw training data. Table 2 contains the mean reconstruction metrics and standard deviation over all test subjects for the runs with the best validation performance, and Fig. 5 shows the mean reconstruction metrics for all 5 runs.

**Table 2:** Reconstruction metrics for the ablation experiments (mean ± standard deviation over all 20 test subjects). Experiments used a combination of real, sinusoidal, and synthetic phase maps, and real and synthetic coil sensitivity maps (CSM). Real maps were sourced from the raw data, whereas synthetic maps were generated from magnitude-only images (marked with * to indicate this difference in data type)

Metric/Experiment	Training scans			
	20	50	100	160
**MAE**				
Baseline	0.0124 ± 1.34e-3^‡^	0.0106 ± 1.11e-3^‡^	0.0102 ± 1.20e-3^†^	0.0101 ± 0.99e-3^†^
Real phase, real CSM	0.0121 ± 1.28e-3^‡^	0.0106 ± 1.06e-3	0.0103 ± 0.99e-3^†^	0.0103 ± 0.95e-3^†^
Real phase, synth. CSM *	0.0115 ± 1.21e-3	0.0108 ± 1.10e-3^‡^	0.0104 ± 1.03e-3	0.0102 ± 0.95e-3^†^
Sinusoidal phase, synth. CSM *	0.0114 ± 1.25e-3	0.0107 ± 1.14e-3^‡^	0.0106 ± 1.12e-3^‡^	0.0105 ± 1.11e-3^‡^
Synth. phase *, real CSM	0.0118 ± 1.19e-3^‡^	0.0108 ± 1.06e-3^‡^	0.0102 ± 0.95e-3^†^	0.0102 ± 0.92e-3^†^
Synth. phase *, synth. CSM *	0.0114 ± 1.15e-3	0.0105 ± 1.00e-3	0.0104 ± 0.99e-3	0.0104 ± 0.97e-3
**RMSE**				
Baseline	0.0174 ± 2.63e-3^‡^	0.0148 ± 2.07e-3	0.0141 ± 1.94e-3^†^	0.0138 ± 1.74e-3^†^
Real phase, real CSM	0.0178 ± 2.73e-3^‡^	0.0148 ± 2.16e-3	0.0144 ± 2.05e-3	0.0140 ± 1.65e-3
Real phase, synth. CSM *	0.0165 ± 2.40e-3^‡^	0.0153 ± 2.26e-3^‡^	0.0145 ± 1.92e-3	0.0139 ± 1.56e-3^†^
Sinusoidal phase, synth. CSM *	0.0162 ± 2.46e-3	0.0153 ± 2.62e-3^‡^	0.0150 ± 2.44e-3^‡^	0.0147 ± 2.37e-3^‡^
Synth. phase *, real CSM	0.0172 ± 1.99e-3^‡^	0.0151 ± 1.98e-3^‡^	0.0141 ± 1.74e-3^†^	0.0138 ± 1.45e-3^†^
Synth. phase *, synth. CSM *	0.0162 ± 1.86e-3	0.0147 ± 1.85e-3	0.0144 ± 1.82e-3	0.0141 ± 1.49e-3
**SSIM**				
Baseline	0.968 ± 7.48e-3^‡^	0.975 ± 6.00e-3	0.977 ± 5.89e-3^†^	0.977 ± 5.76e-3^†^
Real phase, real CSM	0.969 ± 7.20e-3^‡^	0.976 ± 5.89e-3	0.977 ± 5.88e-3^†^	0.977 ± 5.69e-3^†^
Real phase, synth. CSM *	0.971 ± 6.81e-3	0.975 ± 6.15e-3^‡^	0.976 ± 6.22e-3	0.977 ± 5.86e-3^†^
Sinusoidal phase, synth. CSM *	0.972 ± 6.72e-3^†^	0.975 ± 6.20e-3	0.976 ± 6.11e-3^‡^	0.976 ± 5.88e-3^‡^
Synth. phase *, real CSM	0.969 ± 7.13e-3^‡^	0.975 ± 6.17e-3^‡^	0.977 ± 5.81e-3^†^	0.977 ± 5.79e-3^†^
Synth. phase *, synth. CSM *	0.971 ± 6.98e-3	0.975 ± 6.28e-3	0.976 ± 6.08e-3	0.977 ± 5.89e-3

In comparison to the proposed method (Synthetic phase and synthetic CSM), values marked with † were significantly better (*P* < 0.05) and values marked with ‡ were significantly worse (*P* < 0.05), in a paired t-test (paired per test subject)

For small training set sizes (20 and 50 scans), all tested alternatives were equal or significantly worse (*P* < 0.05) than the proposed method (synthetic phase and synthetic CSM), whereas for large training set sizes (100 and 160 scans) all except the experiment using sinusoidal phase were equal or significantly better (*P* < 0.05) than the proposed method. This is consistent with the main results presented in Table 1 and Fig. 5, which showed that training with real raw data was preferable when enough data is available, whereas with less data the additional variance introduced by randomly generating phase and CSM maps was preferable. The relative minor difference in the mean errors demonstrates that the performance of the synthetic data generation was close to the performance of the real, ground-truth maps.

The differences between the baseline experiment and regenerating raw data from the real phase and CSM maps show that some information was changed or lost in the process of creating phase and CSM maps from raw data, for example by filtering underlying noise. Naturally, this also affected the synthetic data experiments and suggests room for improvement in how the raw data is calculated from magnitude, phase, and coil sensitivity maps.

Using sinusoidal phase patterns showed results comparable with our proposed methodology with limited training data (20 and 50 scans), but substantially worse results when more training data was available (100 and 160 scans), indicating the benefit of using deep learning to generate phase patterns that are more realistic than simple geometric functions.

**Fig. 7:**
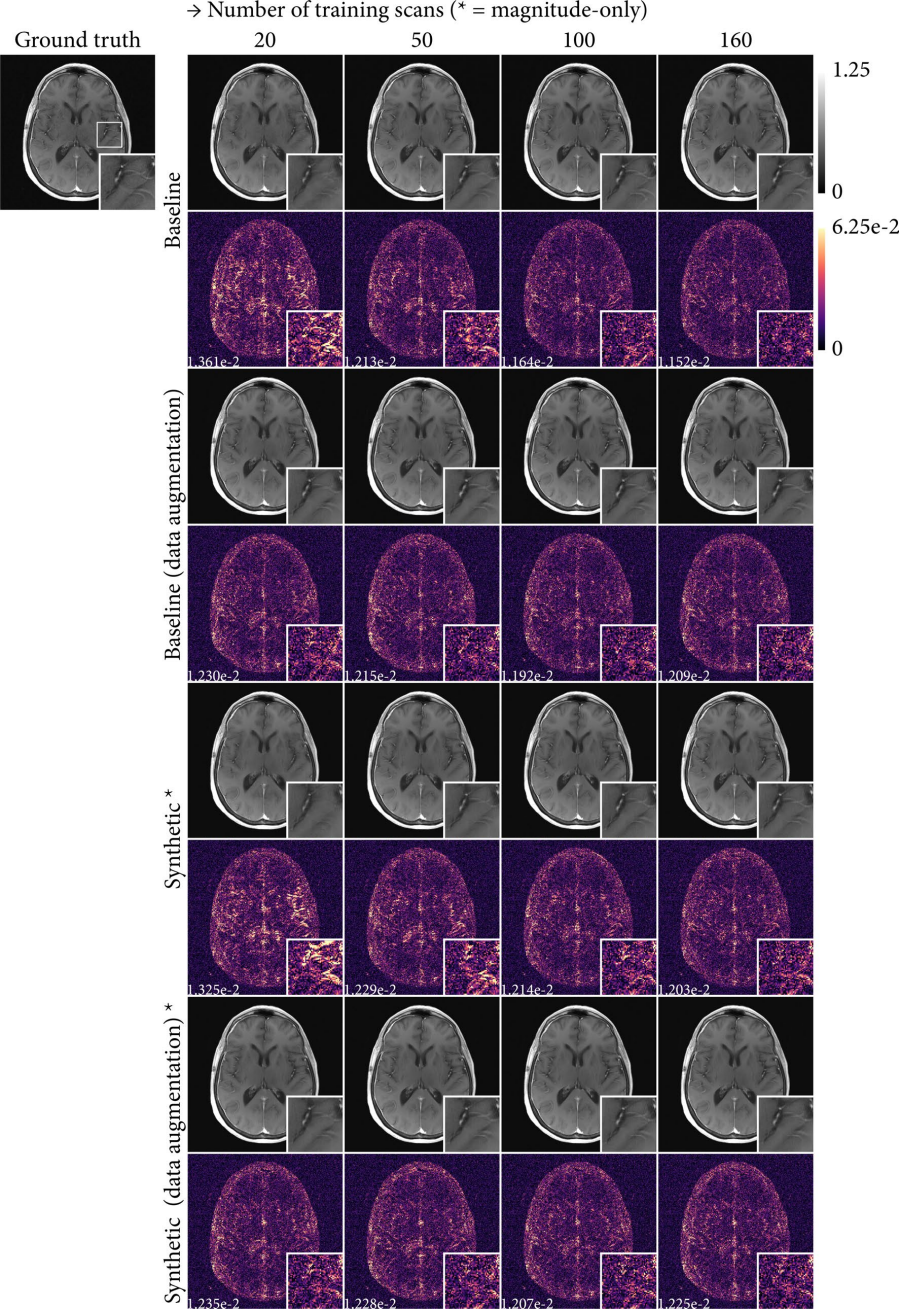
Reconstructed images for all of the experiments (odd rows) with increasing training data (columns, 20, 50, 100, and 160 scans) for one representative slice of the test set. Additionally, absolute error maps for each image relative to the ground truth (top left) are shown (even rows). For each error map, the mean absolute error (MAE) over the entire slice is annotated. A zoomed region (indicated on the ground truth image) highlights varying reconstruction quality in fine-grained image features. Important to note that the variable amounts of training data for the synthetic data experiments were magnitude-only images (marked with *) in addition to real raw data from 20 images (e.g. the experiment with 160 scans used 20 real raw images and 140 magnitude-only images, see Sect. "Experimental setup"). In contrast, the baseline experiments used variable amounts of real raw data

Figure 7 shows reconstructions of one representative slice from the test dataset for the run with the best validation score for each of the experiments performed in this study. In general, the reconstruction errors follow the global reconstruction metrics presented in Fig. 5, yielding smaller errors with increasing amounts of training data. However, the error maps show that reconstruction errors vary spatially among experiments (i.e. not all regions improved homogeneously). This illustrates the difficulty with evaluating images using image-wide reconstruction metrics, a well-known issue in the development of accelerated MR imaging [46].

## Discussion

In this study, we investigated the effects of limited data on deep-learning-based MR image reconstruction and explored how synthetic raw data generation can mitigate these effects. Our methodology for generating synthetic raw data from magnitude images showed improvements in reconstruction quality with an increasing number of magnitude images. This was similar to the improvement observed when increasing the amount of real raw data. As magnitude images tend to be more readily available, this lowers the data burden for training deep-learning-based MRI reconstruction methods.

In comparing our experiments, it is important to remember that the synthetic data was based solely on magnitude images, which otherwise would not be useable for training DL-based MR image reconstruction. As such, the slightly better performance in the baseline experiment with more data is not unexpected. Once a sufficient quantity of data is available, it is unreasonable to expect that models trained on synthetic raw data will perform as well as those trained on real raw data.

In this study, we did not investigate the impact of any post-processing done on the magnitude images. In MR image reconstruction, magnitude images are often processed with a ringing filter and/or interpolated to a higher resolution. For storage, the images are quantized and sometimes thresholded to suppress background noise. Because many of these filters are irreversible, this may impact the quality of synthetic raw data generated from post-processed magnitude images, which will need to be further investigated.

All experiments showed variance across 5 runs of the experiment due to random initialization of the networks and random sampling of the training data. Especially with smaller training sets there was substantial variance in the overall reconstruction metrics (Fig. 5). This suggests that it is important to find a good result using repeated runs (e.g. using a validation set), or that improvements in the stability of training these networks may be needed. This variance was lower for the experiments involving synthetic data, which suggests that the increased variability in the synthetic raw data reduced the impact of random sampling of training data, and therefore improved the robustness of training.

### Data augmentation

The effectiveness of data augmentation has been widely established, both in deep learning in general [18], as well as DL-based MR image reconstruction [17], and our baseline experiments confirmed this for smaller amounts of training data with both real and synthetic data (20 and 50 scans). Therefore, data augmentation should be applied when training data is limited. However, for larger training sets we found that data augmentation may have an adverse effect on reconstruction quality. This is most likely related to the data augmentation parameters, i.e. the frequency and magnitude with which the augmentations are applied. With more training data available, applying less data augmentation will yield better performance. However, finding optimal parameters for augmentation is computationally expensive, because evaluating the performance requires a full training run for each set of parameters [47].

### Synthetic raw data

When using synthetically generated raw MRI training data, we observed reduced reconstruction errors with increasing amounts of magnitude data. Even at 160 scans (the maximum available in this study) we still observed improvement, and it should be investigated whether this trend continues when more magnitude data is available. While the results at 160 scans using synthetic data were slightly worse than the baseline experiment (~ 2.6% MAE) and equivalent to the baseline with data augmentation experiment (less than 0.1% MAE difference), this was achieved in a fundamentally different way using only magnitude data, whereas the baseline experiments used additional real raw data. Therefore, some gap in performance is expected and indicative of a difference between synthetic and real raw data.

In comparison to the recent results presented by Deveshwar et al. [25] for generating phase maps from magnitude data, our phase maps showed very few artifacts, which was likely achieved by avoiding the image-based adversarial losses used in that study. Furthermore, our adversarial auto-encoder approach was capable of generating many phase maps from the same magnitude image, showed no artifacts in the generation of multi-coil coil-sensitivity maps, and training the synthesis networks was both fast and robust, with a total training time of 9.3 h.

A small number of phase maps showed discontinuities, which arose from interpolating in latent space. The raw data generated from these samples would be outside the distribution of real raw data, but nonetheless presented a valid reconstruction problem. As such, we expect that the only impact of this broadening of the training distribution would be a possible slower convergence of the reconstruction network.

Enabling the use of magnitude data in DL MR reconstruction experiments may permit broader use of existing databases (e.g. research, biobanks, and/or hospital), which were previously not suitable for training DL-based MR reconstruction networks. Augmenting such databases with small quantities of raw data to allow for the training of synthesis networks would retrospectively make the whole database available for MR reconstruction experiments. This may reduce the burden for prospective collection and storing of raw data.

With sufficient magnitude data available, synthetic data generation provides a promising alternative to acquiring similar amounts of real raw data, with only slightly degraded performance. Combining this approach with data augmentation yielded an additional benefit, but only when training with limited amounts of training data. An important difference between synthetic data and data augmentation is that synthetic data does not necessarily broaden the distribution of the data, because the variance in the generated maps is learned from real data, whereas data augmentation may introduce unrealistic transformations (e.g. too large rotations). Furthermore, because DL-based synthetic data generation is data-driven, it has fewer manually chosen parameters than data augmentation, which makes it easier to apply in a robust manner.

Our approach of separately synthesizing phase and coil sensitivities is a straightforward way to constrain the synthetic raw data and allowed training of the synthesis networks with limited amounts of training data. The proposed bi-directional conditional adversarial auto-encoder architecture proved to be capable of modeling variability in the phase and CSM maps. This is especially notable considering that for each magnitude image only one ground truth map was available, i.e. there was no explicit information on the variability of these maps. The bi-directional nature of our auto-encoder architecture was crucial for achieving this variability, as it ensures that a generated map can be transformed back into its latent vector, establishing a one-to-one relationship between a magnitude image and latent vector, and an output map. This promotes the generation of more diverse synthetic maps by preventing multiple latent vectors from creating the same output maps.

It may appear that the very low dimensionality of the latent space used in the synthesis networks also limits the number of phase and CSM patterns that could be synthesized. However, even with a small latent space, greatly differing patterns (Fig. 4) can be synthesized because additional information is available from the magnitude-only input images and because the synthesis networks are highly non-linear. A larger dimensionality of the latent space can be chosen when more raw data is available for training. Because of the limited dimensionality of the latent space and the fact the synthesis networks operate on a lower resolution, fine-grained details can not be synthesized by the AAE network. However, the application of the super-resolution networks after the initial, low-resolution synthesis succesfully enabled the synthesis of such details based on the high-resolution magnitude images.

A limitation of the synthetic data generation networks applied in this study was the use of a fixed image dimension of $320\times 320$ voxels (and fixed downsampling to $80\times 80$ voxels). This is a common limitation in applying DL on images, which may need to be resolved for synthetic data generation and DL-based MR image reconstruction to be more widely applicable. One possible approach would be to leverage the UNet architecture, which could be trained to operate on variable image dimension.

Our raw data synthesis methodology is scan-specific, and therefore new synthesis networks need to be trained for each scan type, anatomy, and coil geometry. These networks may exhibit some limited generalization to magnitude images from the same anatomy, but acquired with a different sequence type. However, it is unlikely that a phase generation network trained on the brain will provide meaningful phase maps on knee data, for example. Better generalization across scan parameters could be achieved by training the networks with a broader dataset and providing the relevant scan parameters (e.g. echo time) as an additional input to the network.

An important limitation of this study is that we only explored training raw data generation and reconstruction networks with a single scan type, anatomy, and undersampling pattern. This differs from the experimental setup of the FastMRI challenge and the end-to-end variational network benchmark model [13, 44], which trained a single reconstruction network on the full dataset with multiple scan types. However, our approach is more representative of research applications involving new sequences, particularly those with novel contrasts and undersampling strategies (e.g. non-Cartesian encoding) that are not represented in the FastMRI dataset. Although multiple synthesis networks would need to be trained to generate synthetic raw data for multiple scan types, this data can then be combined into a single, large, synthetic dataset that could be competitive with state-of-the-art performance as reported on the full FastMRI dataset, but without requiring thousands of raw datasets and using primarily magnitude data to train.

There may be alternative approaches to generating synthetic raw data that we did not explore in this study. For example, one possibility would be to apply the transformation straight from magnitude images to raw data. Another possibility would be to use unconditional generation, where the raw data generation does not require a magnitude image as input. Finally, although our adversarial auto-encoder provided good results, alternative generative models exist that may have similar or better performance, such as variational auto-encoders [48], optionally combined with the GAN architecture [49], among others. Recently, denoising diffusion probabilistic models [50] have been introduced and show great potential for generative deep learning applications in general (e.g. [51–53]), as well as MR image reconstruction (e.g. [54–56]), while avoiding issues that can occur with GAN implementations, such as mode collapse [57]. However, we expect that more training data would be needed to train such variations of the synthesis networks. This proof of concept study did not aim to compare such alternative methods. Our results show that the AAE model was suitable for synthetic data generation, but we do not claim that an AAE is the optimal approach.

### Conclusion

Exploring the effects of limited training data availability remains an important area of research for DL-based MR reconstructions. For new applications of such reconstruction methods, one cannot simply assume that there will be hundreds of scans available for training, and this study shows that both data augmentation and DL-based synthetic raw data generation are valuable tools in mitigating this lack of training data.

The methods described in this study alleviated the reduction in reconstruction quality associated with limited data availability, but should by no means be considered exhaustive. The rapid development of deep learning methods provides many new opportunities to synthesize increasingly realistic training data, particularly when combined with the underlying MR physics and physiology.

By utilizing deep learning not just in image reconstruction, but also in data processing and synthesis, we can make sure to get the most out of the raw data that is available and allow other data sources to be utilized in ways that were previously not possible.

## Figures and Tables

**Fig. 8:**
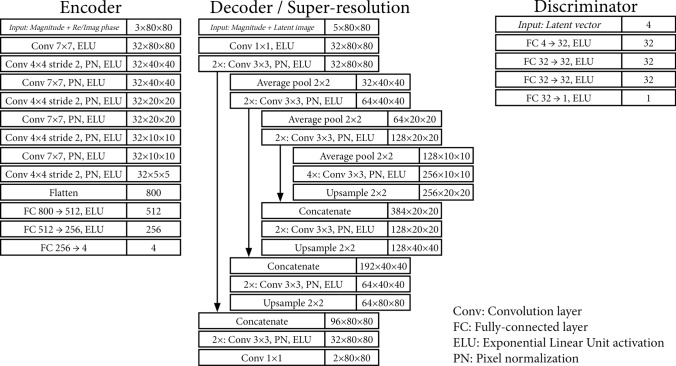
Network architectures are used in the generative and super-resolution networks (Fig. 1). Each network is shown as a list of layers and output shapes (top to bottom). The encoder network is a combination of a fully convolutional network and multi-layer perceptron (MLP) to process 2D maps into a 4-element vector. The decoder and super-resolution network is a standard UNet [59]. The discriminator network is a 3-layer MLP. The input shapes are shown for the auto-encoder for phase maps. For the coil sensitivity maps (CSM) the number of channels is 33 for the encoder input and 32 for the decoder output. For the super-resolution network, the input shape is 3 × 320 × 320 for phase maps and 33 × 320 × 320 for CSM, and the output shape is 2 × 320 × 320 for phase maps and 32 × 320 × 320 for CSM

## Data Availability

The source code used to produce the findings in this study has been made available on GitHub: https://github.com/FrankZijlstra/mri_raw_data_generation/. An interactive demonstration of the generation of phase maps, coil sensitivity maps and raw data is linked and available to run both locally and on Google Colaboratory.

## Implementation details

### Bi-directional conditional adversarial auto-encoder

Our bi-directional conditional adversarial auto-encoder is an extension of the adversarial autoencoder (AAE) architecture [42]. This includes two additions:

1. Our model is conditional on the magnitude image. Rather than just finding a latent-space representation of a phase map, it does so given the magnitude image. This simply means that in evaluating the encoder the magnitude image is concatenated to the phase map, and in evaluating the decoder the magnitude image is concatenated to the latent vector after it is expanded to image space. As such, the latent vector only needs to represent the variation in the phase map that cannot be derived from the magnitude image, allowing it to be low-dimensional.
2. To prevent the model from always generating the same phase map based on the magnitude image while ignoring the latent vector, our model includes an additional reconstruction loss during training. This loss is calculated by first decoding a random latent vector to a phase map, and then encoding it back to a latent vector. The reconstruction loss is then calculated on the difference between the original random latent vector and the encoded latent vector. By ensuring the latent vector can be re-encoded from the generated phase map, our model ensures that the transformation from latent vector to phase map is unique and promotes variance in generated phase maps.

This architecture is shown in Fig. 1A. Furthermore, to improve performance and stability, we apply this model on fourfold downsampled images. To recover the high-resolution images, we trained a super-resolution network, as shown in Fig. 1B.

The network architectures we used were as follows (shown in Fig. 8):

- Encoder: A combination of a convolutional network with a fully connected MLP, based on LeNet [58]
- Decoder: UNet [59]
- Discriminator: MLP with 3 hidden layers
- Super-resolution network: UNet (identical to decoder)

Pixel normalization [60] was used in all networks, except the discriminator, to avoid diminishing gradients.

The bi-directional conditional AAE was trained by optimizing two loss functions:

$$L_{G} = \lambda_{rec}\,||G\left( {z_{enc} ,m} \right) - p|| + \lambda_{z} \,||E\left( {G\left( {z,m} \right),m} \right) - z|| + \lambda_{D}\, BCE\left( {D\left( {z_{enc} } \right),1} \right)\;\;\;with\, z_{enc} = E\left( {p,m} \right)$$

$$L_{D} = BCE\left( {D\left( z \right),1} \right) + BCE\left( {D(z_{enc} } \right),0)$$

Here, ${L}_{G}$ is the generation loss and ${L}_{D}$ the adversarial loss on the discriminator output, *E* is the encoder network, *G* is the decoder network, *D* is the discriminator network, *m* is the magnitude image, *p* is the phase image, *z* is the latent vector, ${z}_{enc}$ is the encoded latent vector, and *BCE* is the binary cross-entropy function [61]. ${\lambda }_{rec}$ was set to 100, ${\lambda }_{z}$ and ${\lambda }_{D}$ were set to 1.

The losses were optimized with an Adam optimizer [62], with a weight decay of 1e-5. Training steps for ${L}_{G}$ and ${L}_{D}$ were alternated. The learning rate was 1e-4 for 50 epochs, followed by a linear decay from 1e-4 to 1e-10 over another 50 epochs. Each epoch was defined as 2000 batches of images with batch size 4.

### Super-resolution network

The super-resolution network used the same UNet architecture as the decoder network. The network was conditional on the high-resolution magnitude image, and the low-resolution input images were upsampled to the high resolution using bilinear interpolation.

For phase maps, the number of input channels was 3 (magnitude + real/imaginary), and the number of output channels was 2. For CSM, the number of input channels was 33 (magnitude + real/ imaginary per receive channel), and the number of output channels was 32.

The super-resolution network loss function was an L1 loss between the super-resolution output and the real high resolution maps. The losses were optimized with the same optimizer settings that were used in training the auto-encoder.
